# MEF: Multidimensional Examination Framework for Prioritization of COVID-19 Severe Patients and Promote Precision Medicine Based on Hybrid Multi-Criteria Decision-Making Approaches

**DOI:** 10.3390/bioengineering9090457

**Published:** 2022-09-08

**Authors:** Karrar Hameed Abdulkareem, Mohammed Nasser Al-Mhiqani, Ahmed M. Dinar, Mazin Abed Mohammed, Mustafa Jawad Al-Imari, Alaa S. Al-Waisy, Abed Saif Alghawli, Mohammed A. A. Al-Qaness

**Affiliations:** 1 College of Agriculture, Al-Muthanna University, Samawah 66001, Iraq; 2 College of Engineering, University of Warith Al-Anbiyaa, Karbala 56001, Iraq; 3 Center for Advanced Computing Technology, Faculty of Information and Communication Technology, Universiti Teknikal Malaysia Melaka, Malacca 75300, Malaysia; 4 Computer Engineering Department, University of Technology- Iraq, Baghdad 19006, Iraq; 5 College of Computer Science and Information Technology, University of Anbar, Anbar 31001, Iraq; 6 Department of Medical Laboratory Techniques, Al-Mustaqbal University College, Hillah 51001, Iraq; 7 Computer Technologies Engineering Department, Information Technology Collage, Imam Ja’afar Al-Sadiq University, Baghdad 10064, Iraq; 8 Computer Science Department, College of Sciences and Humanities, Prince Sattam Bin Abdulaziz University, Aflaj 16278, Saudi Arabia; 9 College of Physics and Electronic Information Engineering, Zhejiang Normal University, Jinhua 321004, China; 10 Faculty of Engineering, Sana’a University, Sana’a 12544, Yemen

**Keywords:** COVID-19, precision medicine, prioritization, Multidimensional Examination Framework, hybrid multi-criteria decision-making, CRITIC, VIKOR

## Abstract

Effective prioritization plays critical roles in precision medicine. Healthcare decisions are complex, involving trade-offs among numerous frequently contradictory priorities. Considering the numerous difficulties associated with COVID-19, approaches that could triage COVID-19 patients may help in prioritizing treatment and provide precise medicine for those who are at risk of serious disease. Prioritizing a patient with COVID-19 depends on a variety of examination criteria, but due to the large number of these biomarkers, it may be hard for medical practitioners and emergency systems to decide which cases should be given priority for treatment. The aim of this paper is to propose a Multidimensional Examination Framework (MEF) for the prioritization of COVID-19 severe patients on the basis of combined multi-criteria decision-making (MCDM) methods. In contrast to the existing literature, the MEF has not considered only a single dimension of the examination factors; instead, the proposed framework included different multidimensional examination criteria such as demographic, laboratory findings, vital signs, symptoms, and chronic conditions. A real dataset that consists of data from 78 patients with different examination criteria was used as a base in the construction of Multidimensional Evaluation Matrix (MEM). The proposed framework employs the CRITIC (CRiteria Importance Through Intercriteria Correlation) method to identify objective weights and importance for multidimensional examination criteria. Furthermore, the VIKOR (VIekriterijumsko KOmpromisno Rangiranje) method is utilized to prioritize COVID-19 severe patients. The results based on the CRITIC method showed that the most important examination criterion for prioritization is COVID-19 patients with heart disease, followed by cough and nasal congestion symptoms. Moreover, the VIKOR method showed that Patients 8, 3, 9, 59, and 1 are the most urgent cases that required the highest priority among the other 78 patients. Finally, the proposed framework can be used by medical organizations to prioritize the most critical COVID-19 patient that has multidimensional examination criteria and to promptly give appropriate care for more precise medicine.

## 1. Introduction

The global COVID-19 epidemic outbreak has a serious impact on people’s daily life and healthcare systems. To stop the spread of this epidemic, the entire globe has struggled and fought [1]. Therefore, the growing cases of COVID-19 present a new issue to the healthcare system, together with the expected daily service delivery, where it is expected that the rate of death would be significant and the diagnosis procedure will take a long time [2]. The early diagnosis and isolation of infected people are crucial in combating the COVID-19 pandemic. Furthermore, it is important to make sure that the right response is provided for critical cases in order to decrease the mortality rate [3].

Medically, examination criteria such as C-reactive protein (CRP) levels can be employed in the early diagnosis of pneumonia, and patients presented with severe pneumonia had high CRP levels [4]. Additionally, shortness of breath is caused by hypoxia, which is defined as SpO_2_ 90%. However, with COVID-19, the normal level of SpO_2_ is decreased and may even drop to 70%, 60%, or 50%, even if the patient did not have a feeling of being out of breath. An important component in understanding and managing patient care is SpO_2_. It compares how much hemoglobin is currently bound to oxygen to how much hemoglobin remains unbound. A pulse oximeter, a non-invasive medical gadget, is placed over a person’s finger to measure blood oxygen saturation. It is commonly utilized in a hospital’s intensive care unit (ICU), operation unit, and postoperative ward. Other examinations, such as normal WBC or low total WBC, high C-reactive protein (CRP), neutrophil, lymphocyte ratio, lymphopenia, bilateral pneumonia in CXR, low procalcitonin, ground-glass opacity (GGO), significant elevation of D-dimer, serum ferritin level, and crazy paving appearance in chest CT scan, indicate the existence of COVID-19 [5]. Dry cough, fever, headache, tiredness, sore throat, vomiting, sneezing, dyspnea, myalgia, nasal congestion, and rhinorrhea are the most common critical symptoms. Critical consequences such as cardiac damage, pulmonary edema, septic shock, and acute renal injury occur in patients with severe COVID-19 infection [6,7].

All of these criteria are crucial in monitoring a COVID-19 patient’s status, but the large number of these indicators poses a challenge for doctors and emergency services to decide or predict the patients’ severity [8]. Furthermore, the old and those suffering from chronic diseases such as diabetes, cancer identification, chronic respiratory disease, cardiovascular disease, and chronic respiratory disease are at a higher risk of contracting a serious infection [9]. A new approach of prioritizing services could be a solution to such challenges. However, such difficulties are increased when patients have several examination results, each with a separate set of references. Due to the large number of COVID-19 patients, specifically in the United States, and various European countries, the treatments and healthcare provided to infected individuals should be prioritized [10]. The issues described in the literature have emphasized the challenge of distinguishing between mild and severe health conditions for infected patients in terms of examination criteria specific to each patient [11,12].

Prior studies in a specific disease perspective have demonstrated that precision medicine and health outcome priorities of patients and healthcare professionals can differ [13]. Moreover, some studies have pointed to a disparity between what practitioners understand to be their patients’ priorities and what their patients actually prioritize [14]. Considering the various problems associated with COVID-19 [15], approaches that could triage COVID-19 patients may be able to help prioritize treatments for patients who are at high risk of serious disease. Furthermore, effective prioritization plays critical roles in precision medicine. COVID-19 severity levels are as follows: ordinary, mild, critical, and severe [16]. Severe cases require extra medical energy and attention than minor and regular ones. A high percentage of false-positive severe or critical cases may overload healthcare facilities (i.e., beds in the intensive care unit). Delays in reporting severe or urgent cases might also lead to patients at a higher risk of death receiving delayed care. As a result, finding acute conditions as soon as possible is crucial in order to deploy services and intensify care [17].

This goal could be fulfilled by employing a prioritizing approach that ranks patients and displays the priority they are given based on their situations in order to facilitate the process of finding patients with the most urgent cases. Prioritization is usually used to ensure that care is provided in an appropriate and timely way. As a result, patient prioritizing is mainly concerned with determining which patients can safely wait and which cannot. In accordance with medical principles, the main tool for determining priority should be the patient’s condition. This strategy can improve medical staff’s workflow by allowing them to focus their attention and efforts on providing the best treatment for patients who require immediate care. Other patients can wait at indoor healthcare providers (such as clinics and hospitals) in the meanwhile [18].

Multi-criteria decision-making (MCDM) approaches have played vital roles in patients’ prioritization. Two main approaches are used for this: one for defining the importance of criteria (weighting approach) and another one to state the final rank (ranking approach) for patients based on the identified criteria. The weighting methods are also divided into two directions namely subjective and objective weighting methods. The analytic hierarchy process (AHP) method [18] is used by respiratory experts to set the subjective weights for the biological laboratory examination criteria for COVID-19 patients. However, despite the success of AHP, the weighting procedure has a significant drawback, namely its inconsistency issue, as well as being affected by expert opinion, as there is no guarantee that the expert cannot produce any bias toward any examined criteria. Objective methods such as CRITIC and Entropy consider the variability in the information. The effects of decision-makers in the calculation of weights are eliminated, as objective methods’ weights are assigned by a mathematical method to the criteria [19].

For the purpose of ranking, each of the VIekriterijumsko KOmpromisno Rangiranje (VIKOR) and Technique for Order of Preference by Similarity to Ideal Solution (TOPSIS) methods have been used widely for patients ranking [18,20]. TOPSIS’s guiding premise is the distance between positive and negative answers. In contrast, it ignores the significance of comparing these distances [21]. The VIKOR procedure is compatible with the discrete alternative because it takes into account the most realistic way of dealing with such situations in the real world. The main advantage of VIKOR is that the best alternative can be quickly identified. VIKOR is thus appropriate in circumstances with several alternatives and attributes [22]. VIKOR utilizes an approach that compromises the priority for various response optimizations. This technique simply ranks the various alternatives based on their closeness to the optimal solution and quickly and accurately identifies the best model [23]. Furthermore, the combination of the weighting method and ranking method in one platform is recommended in the medical domain [20,24]. Thus, the proposed work adopted a hybrid approach based on CRITIC and VIKOR methods. The basis of integration is formed when the weights for the criteria are allocated in accordance with CRITIC. VIKOR is suggested for prioritizing patients with COVID-19.

The innovations and contributions of this work can be summarized as follow:

- Creation of a Multidimensional Evaluation Matrix (MEM) based on real data for different COVID-19 patients’ examination characteristics, such as demographic, laboratory findings, vital signs, symptoms, and chronic conditions.
- Based on multidimensional examination criteria and CRITIC (CRiteria Importance Through Intercriteria Correlation) approach, we provide an evaluation for the most examination criteria for sever COVID-19 patients.
- Develop a framework for prioritization of COVID-19 severe patients on the basis of the real data of constructed MEM and hybrid decision-making approaches (CRITIC and VIekriterijumsko KOmpromisno Rangiranje (VIKOR)). Therefore, we can help health authorities prioritize sever patients by considering the results of different examination criteria for patients infected with COVID-19 to provide the desired intensive care facilities and manage the health conditions of patients.

The rest of this paper is organized as follows: Section 2 summarizes the most related studies on AI with COVID-19 diagnosis. A detailed description and an overview of the steps for the proposed multidimensional framework for COVID-19 patients’ prioritization based on the most urgent cases are presented in Section 3. Section 4 presents the results and a discussion of the prioritization framework of the COVID-19 patients based on the CRITIC weighting and VIKOR ranking methods. Section 5 confirms the validity of proposed work results based on a sensitivity analysis. Finally, Section 6 concludes the study findings, and the future direction presented in this section.

## 2. Related Works

Infectious disease modeling is a method that has been used to explore the mechanisms by which diseases spread, forecast the future course of an outbreak, and assess epidemic control strategies [25]. Mathematics has permeated biology in a variety of ways, including statistics in experimental design; pattern-seeking in bioinformatics; models in evolution, ecology, and epidemiology; and much more [26,27].

In addition to applying mathematical models and AI models for assessing and predicting the significance of factors for evaluating healthcare systems, there are several publications in which the evaluation of healthcare systems is performed by using multi-criteria decision-making (MCDM) tools. Utilizing MCDM in prioritization is a popular topic and a complex issue for patients with COVID-19, especially for multidimensional criteria [28]. The term “multidimensional” is used in the sense that no single value is sufficient alone to determine which patients should receive hospital resources. However, this requires building a robust framework that can be adapted [29]. For more clarity, the traditional way of treating people “first come, first served” should not apply during the pandemic in general and COVID-19 specifically [30]. Therefore, prioritizing some factors (multidimensional factors) should be a better approach to consider the large number of patients.

Prioritization methods have solved many challenges, especially when patients have multi-biological laboratory examination results and vital functions called multi-perspective criteria [31,32]. In clinical prioritization, MCDM methods have proven effective in many healthcare data-focused applications. M. Abdel-Basst et al. applied a model to differentiate between COVID-19 and four other viral chest diseases in an uncertain environment, using the viruses’ primary symptoms and CT scans. The proposed model employs the best worst method (BWM) and the Technique for Order of Preference by Similarity to Ideal Solution (TOPSIS) [33].

We next discuss several studies that deal with the aspects that we explained earlier: MCDM, prioritization, and the multidimensional aspect. Moreover, these related works are discussed in terms of the aim of the research, case study utilized, and criteria used, as shown in Table 1.

Recently, Albahri et al. published two detection-based prioritization frameworks. The first study presented a new multi-biological laboratory examination framework for prioritizing patients with COVID-19 based on integrated MCDA methods. The experiment was conducted based on three phases: patient datasets containing eight biological laboratory examination criteria for six patients with COVID-19 were derived and discussed. The analytic hierarchy process (AHP) method was then used by respiratory experts to set the subjective weights for the biological laboratory examination criteria, and in the final phase, the VIekriterijumsko KOmpromisno Rangiranje (VIKOR) method was employed to prioritize patients in the context of individual and group decision-making (GDM). The second study developed a new integrated decision-making framework for prioritizing COVID-19 patients and detecting the health conditions of asymptomatic carriers. The findings suggest and explain the multiple advantages of the proposed framework for detecting/recognizing patients’ health issues prior to discharge. In the evaluation and benchmarking of COVID-19 ML models, M. A. Mohammed et al. developed a methodology for selecting the best COVID-19 diagnostic model based on Entropy and TOPSIS methods, and they evaluated the proposed methodology by using 50 samples from the COVID-19 Chest X-ray Dataset.

On the other hand, MCDM methods have been utilized in non-clinical prisonization. A new TOPSIS MCDM approach and GMDH can identify and select the significant risk factors and continuously monitor death due to COVID-19 [34]. Another work that was recently published talks about the prioritization of activities. In this work, the authors attempted to identify several activities that should not be performed during the pandemic time. Some selected activities were explored in this paper, and their relevance in preventing COVID-19 was also evaluated. These activities were considered criteria [35].

However, as can be concluded from Table 1, we can raise several issues that we must take into account in order to achieve a robust and reliable prioritization system that is beneficial for health systems:

(a) Real data: With the spread of SARS-CoV-2 worldwide, understanding the basic epidemiological parameter values of COVID-19 from real-world data in mega-cities is essential for disease prevention and control [36]. Comparing them with other models depends on simulated data, which need more validation before being adopted [37].
(b) Dependent criteria: Many studies have shown that symptoms and biomarkers are independently associated with in-hospital death and decisions about the severity of cases [38,39].
(c) Multi-perspective (multidimensional): The identification of hospitalized COVID-19 patients at risk for severe deterioration can be performed by using risk scores that combine several factors, including age; sex; and comorbidities, i.e., diseases such as hypertension, diabetes, and cancer or tumor [40,41,42]. Some risk scores also include additional markers of severity, such as low level of oxygen in the blood, clinical symptoms, and biological factors reflecting multi-organ failures [42,43,44,45,46]. However, there are many unresolved interferences between these factors, including both clinical and biological markers [47]; therefore, there is a need for a comprehensive decision matrix that is inclusive of all of the markers mentioned earlier.

Therefore, the main focus of this study was the prioritization of severe COVID-19 patients based on multidimensional examination criteria that were extracted from real data, as well as the determination of how important these criteria are.

## 3. The Proposed Multidimensional Framework for COVID-19 Patients’ Prioritization

**Figure 1:**
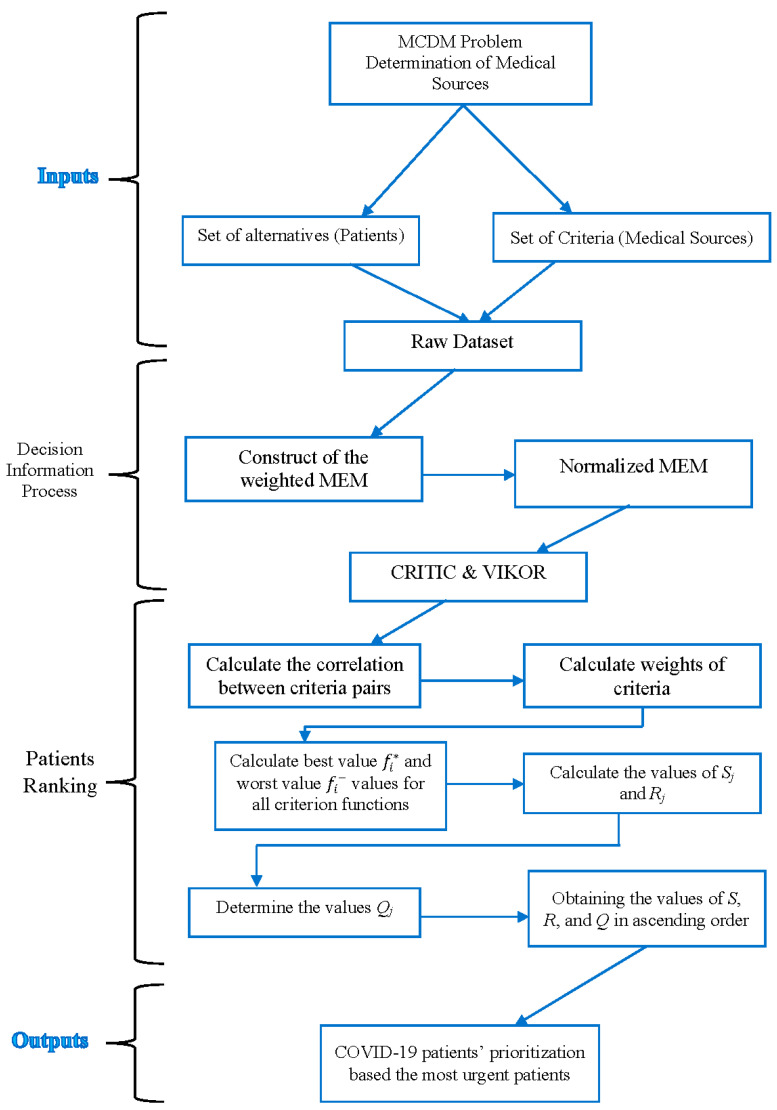
Multidimensional Examination Framework for the prioritization of COVID-19 severe patients.

This section presents a detailed description and an overview of the steps for the proposed multidimensional framework for COVID-19 patients’ prioritization based the most urgent patients. The final output of this proposed framework is patients’ prioritization ranking based on multidimensional medical sources’ criteria used in monitoring COVID-19 patients. The CRITIC weighting method is used to provide criteria weight, while the VIKOR is used for COVID-19 patients’ prioritization ranking. All components of our study are illustrated in the methodology framework in Figure 1.

### 3.1. Construction of Multidimensional Evaluation Matrix (MEM)

**Figure 2:**
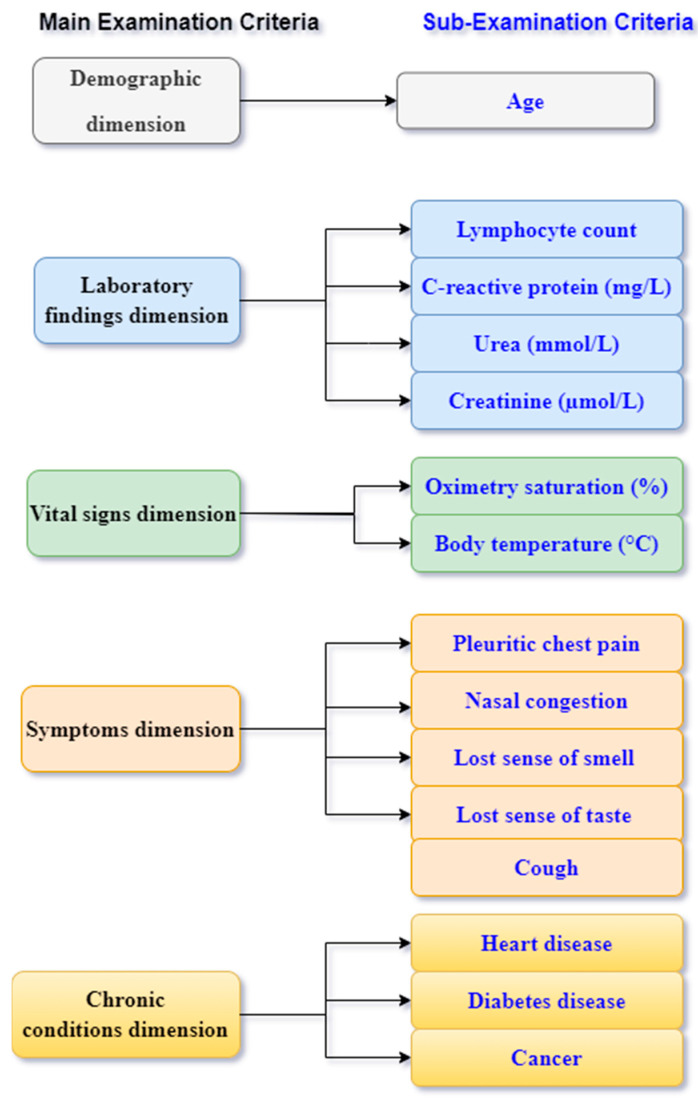
Main and sub-criteria for examination of COVID-19 patients.

The Multidimensional evaluation matrix contains an important element of the proposed framework for COVID-19 patients’ prioritization. It consists of a list of COVID-19 patients, who are considered to be alternatives, and medical sources, which are considered to be the evaluation criteria. The real dataset that was used in our proposed study was extracted from References [8,48]. The total number of people infected with the COVID-19 virus was 78; they were diagnosed under the supervision of specialized doctors and were distributed into Al-Aziziyah Hospital in Wasit Governorate of Iraq. This dataset contains five examination criteria for COVID-19 patients, namely demographic, laboratory findings, vital signs, symptoms, and chronic conditions. Each criterion represents a single dimension in our proposed MEM, as well as MEF. Moreover, each one of these five examination criteria is categorized into sub-criteria, giving a total of 25 sub-criteria. Based on the selected sub-criteria from another study [48], we selected only 15 out of the 25 sub-criteria as the basis for our prioritization process as shown in Figure 2. For instance, the demographic dimension consists of the age of the patients, and the laboratory-findings dimension consists of four sub-criteria, which are lymphocyte count, C-reactive protein (mg/L), urea (mmol/L), and creatinine (µmol/L). The vital-signs dimension consists of two sub-criteria, namely oximetry saturation (%), and body temperature (°C), while the symptoms dimension of the patients consists of five sub-criteria, which are pleuritic chest pain, nasal congestion, cough, lost sense of smell (1/0), and lost sense of taste (1/0). Lastly the chronic-conditions dimension consists of three sub-criteria, which are heart disease, diabetes disease, and cancer. In addition, to summarize the abovementioned scenario, the evaluation criteria in the proposed MEM consist of the main and sub-criteria, which are used to monitor COVID-19 patients from five perspectives. Therefore, a doctor can benchmark the COVID-19 patients according to their urgent situation in consideration of their medical condition. Table 2 illustrates the proposed Multidimensional Evaluation Matrix (MEM) for COVID-19 patients.

### 3.2. Hybridization of CRITIC and VIKOR

The proposed benchmarking method and evaluation methodology were developed by using the techniques of MCDM. Based on the hybridization of the CRITIC weighting method and VIKOR ranking method, this methodology was developed for weighting, ranking, and selecting the most and least critical conditions COVID-19 patients in the proposed matrix for the doctors. The following steps are discussed below. CRITIC and VIKOR are the appropriate methods for ranking and benchmarking of COVID-19 patients. The CRITIC method is used to overcome problems relevant to (1) conflict and trade-off and (2) the encountered evaluation of multi-criteria in the suggested evaluation matrix. VIKOR is often proposed to implement criteria ranking and (3) the value of relational criteria to the suggested matrix of decisions. Consequently, it is justified to combine the CRITIC weighting method and VIKOR ranking method to rank the COVID-19 patients and benchmark them.

#### 3.2.1. Weighting of COVID-19 Examination Criteria Based on CRITIC Method

The CRITIC (CRiteria Importance Through Intercriteria Correlation) method is one of the weighting methods which determines objective weights for criteria. The method was initially proposed by Diakoulaki et al. (1995) [49]. The method was used to derive criterion weights since it tries to determine objective weights of relative relevance in MCDM problems. The proposed approach is based on an analytical examination of the evaluation matrix in order to extract all of the information included in the evaluation criteria. In other words, objective weights are calculated by measuring each evaluation criterion’s intrinsic information. The procedure of determining criteria weights in this method incorporates both the criterion’s standard deviation and its association with other criteria.

An initial evaluation matrix is given in Equation (1), which contains n criteria and m alternatives, where *x_ij_* denotes the performance of the *i*th alternative in relation to the *j*th criterion. The following notations are used to calculate the weight of the *j*th criteria *W_j_*: the quantity of information included in the *j*th criterion is denoted by *C_j_*, the standard deviation of the *j*th criterion is denoted by σ*_j_*, and the correlation coefficient between the *j*th and *k*th criteria is denoted by *rjk*.

The CRITIC method’s calculating stages are shown below based on these notations [50]:

**1st step:** The *X* evaluation matrix is created. It compares the performance of several alternatives based on numerous criteria:

(1) $$X=\lceil x_{ij}\rceil {}_{m*n}=\left[\begin{array}{cc} \begin{array}{c} X_{11}\\ X_{21}\\ X_{31} \end{array} & \begin{array}{c} \begin{array}{ccc} X_{12} & \ldots & X_{1n} \end{array}\\ \begin{array}{ccc} X_{22} & \ldots & X_{2n} \end{array}\\ \begin{array}{ccc} X_{32} & \ldots & X_{3n} \end{array} \end{array}\\ \begin{array}{c} \vdots \\ X_{m1} \end{array} & \begin{array}{c} \begin{array}{ccc} \vdots & \;\;\;\ddots \;\; & \vdots \end{array}\\ \begin{array}{ccc} X_{m2} & \ldots & X_{mn} \end{array} \end{array} \end{array}\right]\;.\;\;\;\;\left(i=1,2,\ldots ,m\;and\;j=1,2,..,n\right).\;$$

The performance value of the *i*th alternative on the *j*th criterion is represented by *xij*.

**2nd step**: The following equation is used to normalize the evaluation matrix:

(2) $$r_{ij}=\frac{x_{ij}-x_{j}^{min}}{x_{j}^{max}-x_{j}^{min}}.\;$$

**3rd step**: Using Equation (3), compute the correlation between criterion pairs:

(3) $$\rho_{jk}=\frac{\sum_{i=1}^{m}\left(r_{ij}-{\bar{r}}_{j}\right)\left(r_{ik}-{\bar{r}}_{k}\right)}{\sqrt{\sum_{i=1}^{m}{\left(r_{ij}-{\bar{r}}_{j}\right)}^{2}\sum_{i=1}^{m}{\left(r_{ik}-{\bar{r}}_{k}\right)}^{2}}}$$

**4th step**:Using Equations (4) and (5), compute the weights of the criterion (5).

(4) $$c_{j}=\sigma_{j}\overset{n}{\underset{k=1}{\sum }}\left(1-\rho_{jk}\right)$$

(5) $$w_{j}=c_{j}/\overset{n}{\underset{k=1}{\sum }}c_{k}$$

where *i* = 1, 2, …, *m;* and *j*, *k* = 1, 2, …, *n.*

#### 3.2.2. Ranking of COVID-19 Severe Patients Based on VIKOR Method

Typically, during the decision-making process, the weighted matrix serves as the starting point for ranking the alternatives. The prior section covers how to obtain the weights that are assigned to each criterion in the EM, giving the weighted EM. During this process, the COVID-19 patients are evaluated and eventually ranked based on the weighted EM. The ranking procedure is as follows:

**1st step**: Define the best $f_{i}^{*}$. value and worst $f_{i}^{-}$ value for all criteria functions:

(6) $$f_{i}^{*}=max_{j}f_{ij},\;f_{i}^{-}=min_{j}f_{ij}$$

where $f_{ij}$. is the *ith* criterion function value for the *x_i_* of alternative. An ideal positive solution increases the benefit criterion while minimizing the cost criteria, whereas the cost criterion is maximized by the ideal negative solution while minimizing the benefit criterion.

**2nd step**: Using the entropy approach, compute the weights of each criterion. A weight set $w=w_{1},\;w_{2},\;w_{3\;},\cdots ,w_{j},\cdots ,\;w_{n}$ from a decision-maker is accommodated in the EM. This set is equal to 1. The following formula can be used to compute the resulting matrix:

(7) $$WM=w_{i}*\frac{f_{i}^{*}-f_{ij}}{f_{i}^{*}-f_{i}^{-}\;}.$$

A weighted EM serves as the output of this step.

**3rd step**: Using the following equations, calculate the values of *S_j_* and *R_j_* (*j* = 1, 2, 3, *…m* and *i* = 1, 2, 3, … *n*):

(8) $$S_{j}=\overset{n}{\underset{i=1}{\sum }}w_{i}*\frac{f_{i}^{*}-f_{ij}}{f_{i}^{*}-f_{i}^{-}\;}$$

(9) $$R_{j}=\underset{i}{\max}w_{i}\;*\frac{f_{i}^{*}-f_{ij}}{f_{i}^{*}-f_{i}^{-}\;}$$

where *S_j_* and *R_j_* denote the measure of the utility and regret for the alternative *f_i_*, and *w_i_* specifies the relative weights of the criterion.

**4th step**: Using the following relation, calculate the values of *Q_j_* (j=(1,2,⋯,J)):

(10) $$Q_{j}=\frac{v\left(S_{j}-S^{*}\right)}{S^{-}-S^{*}}+\frac{\left(1-v\right)\left(R_{j}-R^{*}\right)}{R^{-}-R^{*}}$$

where $S^{*}=\underset{j}{\min}S_{j\;}$, $S^{-}=\underset{j}{\max}S_{j\;}$, $R^{*}=\underset{j}{\min}R_{j\;}$, $R^{-}=\underset{j}{\max}R_{j\;}$, and *v* is defined as the strategy weight of “the majority of criteria” (or “the maximum group utility”) and v = 0.5.

**5th step**: The alternatives can be performed in this step by sorting the values and obtaining the values of *S, R*, and *Q* in ascending order. The lowest value represents the best performance.

**6th step**: The alternative ($a^{\prime }$) is provided as a solution alternative and ranked as the best solution based on the minimal *Q* measure.

**7th step**: Propose alternative *A*^1^, which is ranked the best by the metric *Q* (minimum) if the following two requirements are met, as a compromise solution:

1. C1. Acceptable advantage:

(11) $$Q\left(A^{2}\right)-Q\left(A^{1}\right)⩾DQ$$

where *A*^2^ represents the alternative ranked second in the list by *Q*, *DQ* = 1/(*m* − 1), and *m* is the number of alternatives.

2. C2. Acceptable stability in decision-making:

According to this condition, the alternative *A*^1^, which is best-ranked by *Q*, must also be the best-ranked by *S* or/and *R*.

A set of compromise solutions are suggested if one of the conditions is not met, which consists of the following: alternatives *A*^1^ and *A*^2^ if only condition *C^2^* is not satisfied; or alternatives *A*^1^, *A*^2^, …, *A^M^* if condition *C*1 is not satisfied. *A^M^* is determined by the relation *Q*(*AM*) − *Q*(*A*^1^) < *DQ* for maximum *M* (the positions of these alternatives are “in closeness”).

## 4. Results and Discussions

This section presents the results and discussion of the prioritization framework of the COVID-19 patients based on the CRITIC weighting and VIKOR ranking methods. Section 4.1 describes how the MEM data results were generated from the 78 COVID-19 patients. In Section 4.2, the CRITIC results are discussed, and the VIKOR method results are presented and discussed in detail in Section 4.3.

### 4.1. MEM Results

Table 3 displays the structure of the proposed EM. The top two rows of the table show the five main medical-source criteria, and the second row from the top shows the fifteen sub-criteria, while the first column from the left shows the patients categorized according to their severity; the COVID-19 patients in the list are considered as alternatives in the EM. The values in the EM indicate all the diagnosis data for a total number of 78 people who were infected with the COVID-19 virus and were diagnosed under the supervision of specialized doctors from the Wasit Governorate of Iraq. The 78 patients are divided into three main groups based on the patient’s severity, which was assessed and categorized as being severe, moderate, and mild by doctors according to the Iraqi hospitals’ criteria at admission.

### 4.2. CRITIC Weighting Results

In this section, we discuss the use of the CRITIC method for finding the weights of each criterion. First, the normalized evaluation matrix was determined based on Equation (2); the normalized evaluation matrix is shown in Appendix A. The last row of Appendix A shows the values of standard deviations for all criteria. The values of the correlation coefficient were then calculated, and these are shown in Appendix B. Moreover, the quantity of info (*C_j_*) is shown in Appendix C. Finally, the criteria weights in Table 4 were determined by using Equations (4) and (5).

**Figure 3:**
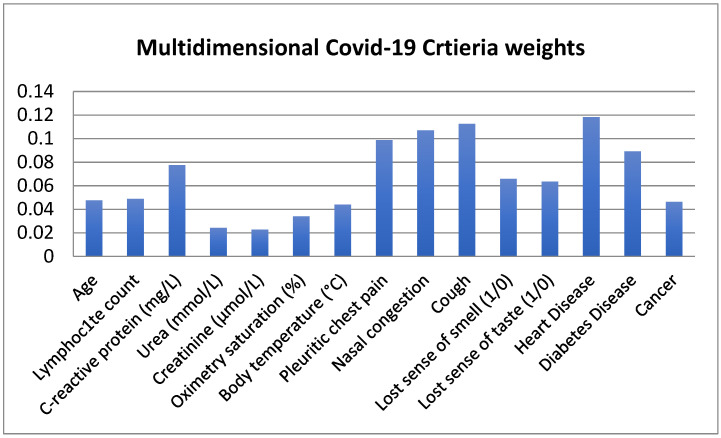
COVID-19 criteria weights.

Based on the result shown in Table 4, Appendix A, Appendix B and Appendix C, and Figure 3, the gained values of *W_j_* and weights of the CRITIC method for the fifteen sub-criteria were obtained by using Equations (4) and (5). Heart disease achieved the maximum CRITIC weight, at 0.118, followed by cough and nasal congestion, with CRITIC weight 0.113 and 0.107, respectively. On the other hand, creatinine achieved the minimum CRITIC weight, at 0.023. Based on the results, the medical source criteria with the highest CRITIC weights are considered to be the most important criteria, whilst the criteria with the lowest CRITIC weight are of least importance.

### 4.3. Ranking Results based on VIKOR Method

This subsection discusses the results of the 78 COVID-19 patients ranking based on the weighted EM. The weighted EM can be obtained by using Equations (5) and (6) and is shown in Table 5. Notably, we considered the age of the patients, lymphocyte count, C-reactive protein (mg/L), urea (mmol/L), creatinine (µmol/L), body temperature (°C), pleuritic chest pain, nasal congestion, cough, lost sense of smell (1/0), lost sense of taste (1/0), heart disease, diabetes disease, and cancer as benefit criteria, while oximetry saturation (%) is considered as cost criteria. Then Equations (7) and (8) were used to calculate alternative distances from the positive ideal solutions and negative ideal solutions. Last, based on Equation (9), the *Qi* values for the 78 COVID-19 patients were calculated. Hence, using VIKOR principles, the 78 COVID-19 patients were ranked based on their urgent situation in consideration of their medical condition. Table 5 shows the result of the weighted EM, while Table 6 displays the final result of VIKOR ranking.

As shown in Table 6, the first five patients, namely Patients 8, 3, 9, 59, and 1, were the most urgent cases that required the highest priority among the other 78 patients, and they obtained minimum values in terms of *Qi*, with 0, 0.116101, 0.125431, 0.136293, and 0.152904, respectively. Therefore, Patient 8 is ranked as the highest priority, and Patients 3, 9, 37, and 1 are next highest priority. Therefore, a rank based on Table 6 was the final outcome of the VIKOR ranking, and the validation processes are based on it.

## 5. Evaluation and Sensitivity Analysis

Numerous authors in their research emphasized that a sensitivity analysis in multi-criteria problems is an indispensable step to confirm the robustness of the obtained solutions [51,52]. Some authors [53,54] suggest checking the robustness of solutions in MCDA problems by changing the input parameters of the model. According to Reference [55], the changing process could be on weight coefficients of the criteria or other parameters into decision-making method. Therefore, our study followed three scenarios to show the robustness of proposed framework in the prioritization of the most critical COVID-19 cases:

**First**: A compromise solution is determined from the alternative that has the best ranking by the measure *Q* (minimum) if the following two conditions are satisfied:

**Condition 1**: Acceptable advantage:

$$DQ=\frac{1}{\left(m-1\right)}=\frac{1}{\left(78-1\right)}=\frac{1}{77}=0.012987013$$

Q(patient 3)−Q(patient 8)=0.116101−0=0.116101

The value of *Q* (*Patient* 3) − *Q* (*Patient* 8) ≤ *DQ*. Thus, the acceptable advantage conditions are satisfied.

**Condition 2**: Acceptable stability in decision-making.

The best ranking results, *Q*, are for *Patient* 8, obtaining the lowest *Q*, *R*, and *S* values with 0, 0.034901, and 0.076941, respectively, as shown in Table 6. So, it can be proved that the condition of acceptable stability in decision-making is satisfied.

Based on the abovementioned details, the two conditions mentioned in Step 7 are satisfied. This can conclude that Patient 8 exhibited the most urgent case and received the highest priority level.

**Figure 4:**
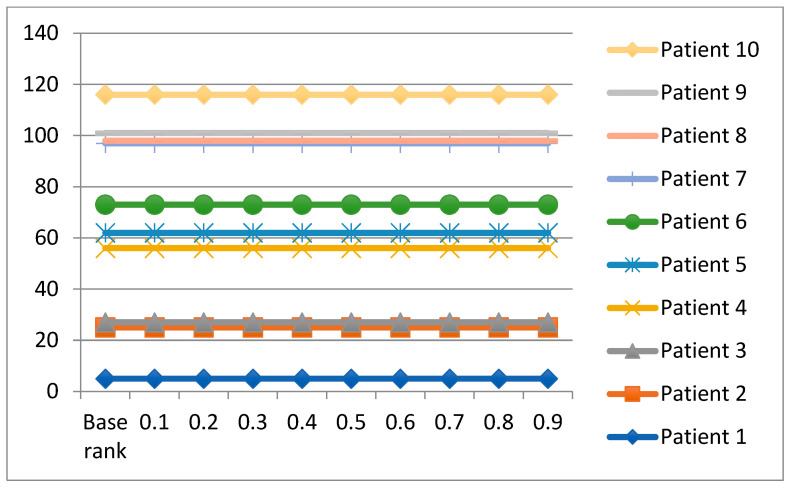
Sensitivity analysis of *V* value for 10 patients.

**Second**: In accordance to Reference [56], changing the value of *V* in the VIKOR method was used to examine the rationality and stability of the proposed framework. Due to the high number of patients in our study, we chose only the first ten patients for the sensitivity analysis as a baseline to verify the ranking of the proposed framework. Furthermore, the *V* value changed nine times, from 0.1 to 0.9, and was compared to the baseline rank, as shown in Figure 4.

As stated before, this work used the disparity of *V* values to validate that none of them impacts the final rank for patients, as shown in Figure 4. The ranking orders of ten patients are the same, and Patient 8 is still the most critical case. Thus, the analysis study can confirm that the results acquired by using the proposed model are reliable and effective. Furthermore, the proposed framework is not sensitive to a change of VIKOR parameters.

**Figure 5:**
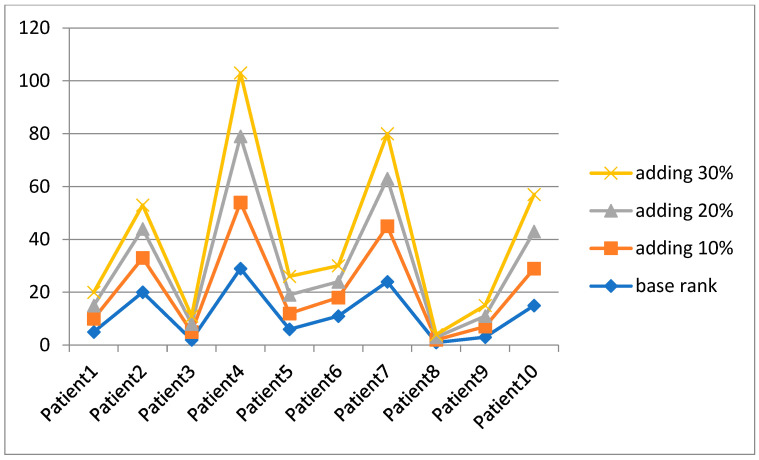
Sensitivity analysis on changing criteria weights by adding (10%, 20%, and 30%).

**Third:** The sensitive analysis based on changing sub-criteria coefficient weights is the third method that was used to verify the proposed framework’s ranking order. In this analysis, we have changed the weight by adding an identified percentage to the original weights. Specifically, all sub-criteria have 10%, 20%, and 30% extra weight than the base weight, as shown in Figure 5.

Based on Figure 5 analysis results, it can be seen there is a high variation into ranking of Patients 2, 4, 6, and 7 but with more high priority rate than base rank. While each of Patients 3, 5, and 9 have very less variation into ranking order with one more high-ranking step. However, Patients 1 and 8 have the same ranking order for three analysis scenarios. Furthermore, still the Patient 8 is the most critical case no matter how the weights change proving the top-ranked patients achieves extraordinarily in different circumstances. It can be verified that the top-ranked-patients results from using the Multidimensional Examination Framework for the prioritization of COVID-19 severe patients is robust and effective. This study can distinguish the priorities of COVID-19 severe patients more easily, and thus help medical staff and decision-makers evaluate and identify the most critical COVID-19 case.

## 6. Conclusions

This study proposed a framework for prioritization of COVID-19 severe patients that have different combination examination criteria that were extracted from different resources. Apart from the existing literature, the MEF has not only considered one dimension of examination factors but multidimensional examination criteria for the prioritization of COVID-19 severe patients such as demographic, laboratory findings, vital signs, symptoms, and chronic conditions.

The five main criteria and fifteen sub-criteria were included in the construction of the multidimensional evaluation matrix. The most effective criteria and sub-criteria for the prioritization of severe COVID-19 patients were identified through given weights by the CRITIC method. Accordingly, COVID-19 patients with different examination criteria were prioritized by using the VIKOR method. From a pool of 78 patients, the proposed intelligent framework succeeded in prioritizing most of the COVID-19 severe patients with different aspects of obtained real data. However, in this study, the authors confirmed several limitations that may be considered for future work. For example, the weighting-based objective method may not consider all environmental conditions; therefore, this may affect the real importance for the examination criteria. Fifteen out of twenty-five sub-criteria were selected based on the Gini Information method for the prioritization of COVID-19 severe patients.

The proposed framework can help health authorities prioritize patients by considering the results not only from a single dimension but from multidimensions. Thus, more complex scenarios are presented for medical staff in order to prioritize the most critical COVID-19 patients. This situation could be even more difficult specifically when critical resources such as beds and ventilators (respirators) in ICUs and medical staff are becoming scarce. Besides that, the proposed methodology can aid healthcare organizations in providing the desired intensive-care services and handling patients’ medical problems. Furthermore, it can help them distinguish among health conditions for large-scale admission process.

However, the number of selected sub-criteria is varied and restricted according to the used mathematical model. Thus, this may affect the selection of the most common examination criteria that consistent of theory and practical domains. When the condition of COVID-19 patients changes, dynamic, more accurate weighting and ranking methods are needed. However, weighting methods that are based on an objective context lack consideration for the changes in a patient’s health circumstance, thus affecting the definition of important examination criteria. Therefore, such methods may not present very accurate ranking for most critical COVID-19 cases. In future work, authors should implement a more robust weighting method based on the integration of subjective and objective contexts to tackle the mentioned issues.

## Figures and Tables

**Table 1:** Related works on COVID-19 identification and prioritization.

Ref.	Aim of the Study	Case Study	Criteria Used	Multidimensional
[33]	Differentiate between COVID-19 and other four viral chest diseases under uncertain environment using the viruses primary symptoms and CT scans	Based on other cases studies	Primary symptoms and CT scans	No
[18]	COVID-19 patient prioritization dependent on their health conditions	Real data of 6 patients from the literature	Laboratory characteristics	No
[20]	Prioritization of asymptomatic carriers	Real data from the literature were extended to 56, using simulated data	Laboratory characteristics	No
[24]	Benchmarking Methodology for Selection of Optimal COVID-19 Diagnostic Model	Real data (publicly available)	CT scans data	No
[34]	Identify and select the significant risk factor and continuous monitoring of death due to COVID-19	Real data (publicly available)	Confirmed and death cases number of COVID-19	No
[35]	Identify some of the suggested activities not to be performed during the pandemic period	Real data (publicly available)	WHO Guidelines	No
Proposed	COVID-19 patient prioritization based on multidimensional criteria	Real data of 78 patients (self-collected or own dataset)	(General factors and comorbidities), (multi-clinical characteristics with SpO_2_ sensor) and (multi-biological characteristics)	Yes

**Table 2:** Proposed Multidimensional Evaluation Matrix (MEM) for COVID-19 prioritization.

	Demographic	Laboratory Findings				Vital Signs		Symptoms					Chronic Conditions		
Patients	Age	Lymphocyte Count	C-reactive Protein (mg/L)	Urea (mmol/L)	Creatinine (µmol/L)	Oximetry Saturation (%)	Body Temperature (°C)	Pleuritic Chest Pain	Nasal Congestion	Cough	Lost Sense of Smell (1/0)	Lost Sense of Taste (1/0)	Heart Disease	Diabetes Disease	Cancer
P1	AV (P1/Ts)	LCV (P1/Ts)	CrPV (P1/Ts)	UrV (P1/Ts)	CrV (P1/Ts)	OSV (P1/Ts)	BTV (P1/Ts)	PCPV (P1/Ts)	NCV (P1/Ts)	CoV (P1/Ts)	LSV (P1/Ts)	LTV (P1/Ts)	HDV (P1/Ts)	DDV (P1/Ts)	CaV (P1/Ts)
P2	AV (P2/Ts)	LCV (P2/Ts)	CrPV (P2/Ts)	UrV (P2/Ts)	CrV (P2/Ts)	OSV (P2/Ts)	BTV (P2/Ts)	PCPV (P2/Ts)	NCV (P2/Ts)	CoV (P2/Ts)	LSV (P2/Ts)	LTV (P2/Ts)	HDV (P2/Ts)	DDV (P2/Ts)	CaV (P2/Ts)
P3	AV (P3/Ts)	LCV (P3/Ts)	CrPV (P3/Ts)	UrV (P3/Ts)	CrV (P3/Ts)	OSV (P3/Ts)	BTV (P3/Ts)	PCPV (P3/Ts)	NCV (P3/Ts)	CoV (P3/Ts)	LSV (P3/Ts)	LTV (P3/Ts)	HDV (P3/Ts)	DDV (P3/Ts)	CaV (P3/Ts)
..….	……	……	……	……	……	……	……	……	……	……	……	……	……	……	……
Pn	AV (Pn/Ts)	LCV (Pn/Ts)	CrPV (Pn/Ts)	UrV (Pn/Ts)	CrV (Pn/Ts)	OSV (Pn/Ts)	BTV (Pn/Ts)	PCPV (Pn/Ts)	NCV (Pn/Ts)	CoV (Pn/Ts)	LSV (Pn/Ts)	LTV (Pn/Ts)	HDV (Pn/Ts)	DDV (Pn/Ts)	CaV (Pn/Ts)

**Table 3:** MEM data.

	Demographic	Laboratory Findings				Vital Signs		Symptoms					Chronic Conditions		
Patients	Age	Lymphocyte Count	C-reactive Protein	Urea	Creatinine	Oximetry Saturation	Body Temperature	Pleuritic Chest Pain	Nasal Congestion	Cough	Lost Sense of Smell	Lost Sense of Taste	Heart Disease	Diabetes Disease	Cancer
1	65	4.1	1	66	1.8	80	38	1	1	1	1	1	1	1	0
2	87	7.5	2	98	2.6	66	39	1	1	1	1	1	0	0	1
3	72	3.6	2	35	0.8	71	39.5	1	1	1	1	1	1	1	0
4	63	5.1	2	30	1	85	40	1	1	1	1	1	0	0	0
5	55	2.1	2	40	0.9	88	39	1	1	1	1	1	1	0	0
6	68	3.8	2	299	21.9	70	39	1	1	1	1	1	0	1	1
7	75	2.3	2	77	1.2	61	40	1	1	1	1	1	0	0	0
8	79	4.1	2	100	3.2	55	40	1	1	1	1	1	1	1	1
9	60	8.5	2	49	1.8	87	38	1	1	1	1	1	1	1	0
10	57	11.2	2	38	0.4	68	39	1	0	1	1	1	1	0	0
11	60	8.3	2	48	0.56	71	38.6	1	0	1	1	1	1	0	1
12	78	0.4	2	87	0.6	82	37	1	0	1	1	1	1	0	0
13	60	1.9	2	31	0.6	84	39	1	0	1	1	1	1	1	0
14	38	0.8	1	25	0.4	86	37	1	1	1	1	1	0	1	0
15	34	0.5	1	31	0.5	90	37	0	0	1	1	1	0	0	0
16	38	5.2	2	35	0.6	80	39	0	0	0	1	1	0	0	0
17	45	0.9	1	37	0.6	84	37	1	0	1	1	1	0	0	0
18	85	0.5	2	85	1.9	87	39	0	1	0	1	1	1	1	0
19	60	2.1	2	50	1	79	38	1	0	1	1	1	1	0	0
20	80	0.5	2	87	2.1	75	39	1	1	0	1	1	1	1	0
21	55	13.3	1	40	0.9	91	37	0	0	1	1	1	0	0	0
22	80	10.7	2	45	1	80	38	1	1	0	1	1	1	0	0
23	55	2.8	1	25	0.3	93	37	0	1	0	1	1	1	0	0
24	60	1.4	1	45	1	84	39	1	0	0	1	1	1	0	0
25	57	3.4	1	28	0.5	87	37	0	1	0	1	1	0	0	0
26	50	4.2	1	31	0.7	90	37	1	0	1	1	1	0	0	0
27	70	3.7	0	22	0.5	82	37	0	0	0	1	1	0	0	0
28	55	1.8	1	40	0.9	92	38	1	0	1	1	1	1	1	0
29	60	2	1	23	0.45	88	38	1	0	0	1	1	0	0	0
30	46	1.4	1	30	0.42	85	38.5	0	1	0	1	1	1	0	0
31	35	1.7	1	33	0.5	90	37	0	0	1	1	1	0	0	0
32	37	1.1	1	37	0.52	66	38	0	1	1	1	1	0	0	0
33	65	1.2	1	24	0.3	80	37	0	1	0	1	1	1	0	0
34	35	1.1	1	22	0.4	83	39	0	1	0	1	1	0	0	0
35	42	1	0	37	0.5	88	37	0	0	1	1	1	0	0	0
36	75	1.3	0	50	0.9	81	38	1	0	0	1	1	1	1	0
37	45	0.8	1	36	0.7	86	39.5	1	0	0	1	1	0	0	0
38	30	2.1	1	38	0.8	90	37	0	0	1	1	1	0	0	0
39	35	1.3	1	44	0.7	88	37	0	1	1	1	1	1	1	0
40	65	1.2	0	36	0.5	80	38	1	0	0	1	1	0	0	0
41	56	1.2	1	50	0.8	89	37	1	1	1	1	1	0	0	0
42	58	0.8	0	64	0.5	81	39	0	1	0	1	1	1	0	0
43	57	1.7	1	38	0.6	88	38	1	1	0	1	1	1	1	0
44	30	1.2	1	30	0.6	87	38.5	1	0	1	1	1	0	0	0
45	46	1	1	39	0.7	90	38.5	1	1	0	1	1	0	0	0
46	40	0.9	1	28	0.4	89	37	1	0	1	1	1	1	0	0
47	35	1.6	1	38	0.55	86	37	0	1	0	1	1	0	0	0
48	60	1.7	1	33	0.3	85	39	1	0	0	0	0	1	1	0
49	65	2.2	1	57	0.86	91	37	0	1	0	0	0	1	1	0
50	60	0.9	0	30	0.7	90	38	0	1	0	0	0	1	0	0
51	44	1.8	1	33	0.9	90	37.5	0	0	1	1	0	0	0	0
52	65	2.8	0	45	0.8	92	38	0	0	1	1	1	1	1	0
53	53	3.4	1	48	1.3	89	38	0	0	1	1	1	0	0	0
54	56	4.1	0	29	1	88	37	0	0	1	1	1	1	0	0
55	49	3.7	0	37	0.7	90	37.8	1	1	1	1	1	1	0	0
56	58	2.9	0	49	0.9	93	37.7	1	0	1	1	1	1	1	0
57	61	3.3	1	45	1.2	91	38.2	0	0	1	1	1	0	1	0
58	67	1.2	0	53	1.9	88	38	1	1	1	1	1	0	1	0
59	71	0.8	1	90	2.9	83	38	1	1	1	1	1	1	1	1
60	40	3.6	0	27	0.6	95	37	0	0	1	1	1	0	0	0
61	74	0.95	0	58	0.9	90	38	1	0	1	1	1	1	0	0
62	66	1.35	0	51	0.9	85	38	1	0	0	1	1	0	0	0
63	65	1.1	0	43	0.7	90	38	1	0	1	1	1	1	0	0
64	27	0.8	0	29	0.4	98	37	0	0	0	1	1	0	0	0
65	45	2.2	0	25	0.5	94	37	0	0	1	1	1	0	0	0
66	25	0.9	0	26	0.3	94	37	0	1	1	1	1	0	0	0
67	50	1.3	0	36	0.4	99	38.5	0	0	1	1	1	0	0	0
68	80	1	0	27	0.8	88	38	1	1	1	0	0	1	0	0
69	55	0.9	0	47	0.8	91	36	0	0	0	0	0	1	0	0
70	20	0.9	0	24	0.7	96	37.7	0	0	0	1	1	0	0	0
71	16	9.1	0	21	0.95	96	35.9	0	0	0	0	1	0	0	0
72	33	3.5	0	33	1.1	98	36	1	0	0	1	1	0	0	0
73	35	4.1	0	35	1.2	99	36	0	0	0	1	1	0	0	0
74	26	2.7	0	26	0.8	95	38	0	0	1	1	1	0	0	0
75	41	3.2	0	21	0.79	95	37.8	1	0	0	1	1	0	0	0
76	46	4.2	0	45	1.2	94	38	1	1	1	1	1	1	0	0
77	13	3.3	0	33	0.9	99	38	0	0	0	0	1	0	0	0
78	22	1.5	0	29	0.93	97	38	0	0	0	1	1	0	0	0

**Table 4:** The criteria weights’ values.

Calculate Weight, W	*W_j_*
Age	0.048
Lymphocyte count	0.049
C-reactive protein	0.077
Urea	0.024
Creatinine	0.023
Oximetry saturation	0.034
Body temperature	0.044
Pleuritic chest pain	0.099
Nasal congestion	0.107
Cough	0.113
Lost sense of smell	0.066
Lost sense of taste	0.063
Heart Disease	0.118
Diabetes Disease	0.089
Cancer	0.046

**Table 5:** Weighted MEM of COVID-19 patients.

	Demographic	Laboratory Findings				Vital Signs		Symptoms					Chronic Conditions		
Patients	Age	Lymphocyte Count	C-reactive Protein	Urea	Creatinine	Oximetry Saturation	Body Temperature	Pleuritic Chest Pain	Nasal Congestion	Cough	Lost Sense of Smell	Lost Sense of Taste	Heart Disease	Diabetes Disease	Cancer
Weight	0.047613	0.048938	0.077444	0.024142	0.022652	0.034111	0.044037	0.098639	0.107028	0.112511	0.065791	0.063479	0.118163	0.089159	0.046293
1	0.014	0.035	0.039	0.020	0.021	0.019	0.021	0.000	0.000	0.000	0.000	0.000	0.000	0.000	0.046
2	0.000	0.022	0.000	0.017	0.020	0.009	0.011	0.000	0.000	0.000	0.000	0.000	0.118	0.089	0.000
3	0.010	0.037	0.000	0.023	0.022	0.012	0.005	0.000	0.000	0.000	0.000	0.000	0.000	0.000	0.046
4	0.015	0.031	0.000	0.023	0.022	0.023	0.000	0.000	0.000	0.000	0.000	0.000	0.118	0.089	0.046
5	0.021	0.042	0.000	0.022	0.022	0.026	0.011	0.000	0.000	0.000	0.000	0.000	0.000	0.089	0.046
6	0.012	0.036	0.000	0.000	0.000	0.012	0.011	0.000	0.000	0.000	0.000	0.000	0.118	0.000	0.000
7	0.008	0.042	0.000	0.019	0.022	0.005	0.000	0.000	0.000	0.000	0.000	0.000	0.118	0.089	0.046
8	0.005	0.035	0.000	0.017	0.020	0.000	0.000	0.000	0.000	0.000	0.000	0.000	0.000	0.000	0.000
9	0.017	0.018	0.000	0.022	0.021	0.025	0.021	0.000	0.000	0.000	0.000	0.000	0.000	0.000	0.046
10	0.019	0.008	0.000	0.023	0.023	0.010	0.011	0.000	0.107	0.000	0.000	0.000	0.000	0.089	0.046
11	0.017	0.019	0.000	0.022	0.022	0.012	0.015	0.000	0.107	0.000	0.000	0.000	0.000	0.089	0.000
12	0.006	0.049	0.000	0.018	0.022	0.021	0.032	0.000	0.107	0.000	0.000	0.000	0.000	0.089	0.046
13	0.017	0.043	0.000	0.023	0.022	0.022	0.011	0.000	0.107	0.000	0.000	0.000	0.000	0.000	0.046
14	0.032	0.047	0.039	0.024	0.023	0.024	0.032	0.000	0.000	0.000	0.000	0.000	0.118	0.000	0.046
15	0.034	0.049	0.039	0.023	0.022	0.027	0.032	0.099	0.107	0.000	0.000	0.000	0.118	0.089	0.046
16	0.032	0.031	0.000	0.023	0.022	0.019	0.011	0.099	0.107	0.113	0.000	0.000	0.118	0.089	0.046
17	0.027	0.047	0.039	0.023	0.022	0.022	0.032	0.000	0.107	0.000	0.000	0.000	0.118	0.089	0.046
18	0.001	0.049	0.000	0.019	0.021	0.025	0.011	0.099	0.000	0.113	0.000	0.000	0.000	0.000	0.046
19	0.017	0.042	0.000	0.022	0.022	0.019	0.021	0.000	0.107	0.000	0.000	0.000	0.000	0.089	0.046
20	0.005	0.049	0.000	0.018	0.021	0.016	0.011	0.000	0.000	0.113	0.000	0.000	0.000	0.000	0.046
21	0.021	0.000	0.039	0.022	0.022	0.028	0.032	0.099	0.107	0.000	0.000	0.000	0.118	0.089	0.046
22	0.005	0.010	0.000	0.022	0.022	0.019	0.021	0.000	0.000	0.113	0.000	0.000	0.000	0.089	0.046
23	0.021	0.040	0.039	0.024	0.023	0.029	0.032	0.099	0.000	0.113	0.000	0.000	0.000	0.089	0.046
24	0.017	0.045	0.039	0.022	0.022	0.022	0.011	0.000	0.107	0.113	0.000	0.000	0.000	0.089	0.046
25	0.019	0.038	0.039	0.024	0.022	0.025	0.032	0.099	0.000	0.113	0.000	0.000	0.118	0.089	0.046
26	0.024	0.035	0.039	0.023	0.022	0.027	0.032	0.000	0.107	0.000	0.000	0.000	0.118	0.089	0.046
27	0.011	0.036	0.077	0.024	0.022	0.021	0.032	0.099	0.107	0.113	0.000	0.000	0.118	0.089	0.046
28	0.021	0.044	0.039	0.022	0.022	0.029	0.021	0.000	0.107	0.000	0.000	0.000	0.000	0.000	0.046
29	0.017	0.043	0.039	0.024	0.022	0.026	0.021	0.000	0.107	0.113	0.000	0.000	0.118	0.089	0.046
30	0.026	0.045	0.039	0.023	0.023	0.023	0.016	0.099	0.000	0.113	0.000	0.000	0.000	0.089	0.046
31	0.033	0.044	0.039	0.023	0.022	0.027	0.032	0.099	0.107	0.000	0.000	0.000	0.118	0.089	0.046
32	0.032	0.046	0.039	0.023	0.022	0.009	0.021	0.099	0.000	0.000	0.000	0.000	0.118	0.089	0.046
33	0.014	0.046	0.039	0.024	0.023	0.019	0.032	0.099	0.000	0.113	0.000	0.000	0.000	0.089	0.046
34	0.033	0.046	0.039	0.024	0.023	0.022	0.011	0.099	0.000	0.113	0.000	0.000	0.118	0.089	0.046
35	0.029	0.047	0.077	0.023	0.022	0.026	0.032	0.099	0.107	0.000	0.000	0.000	0.118	0.089	0.046
36	0.008	0.046	0.077	0.022	0.022	0.020	0.021	0.000	0.107	0.113	0.000	0.000	0.000	0.000	0.046
37	0.027	0.047	0.039	0.023	0.022	0.024	0.005	0.000	0.107	0.113	0.000	0.000	0.118	0.089	0.046
38	0.037	0.042	0.039	0.023	0.022	0.027	0.032	0.099	0.107	0.000	0.000	0.000	0.118	0.089	0.046
39	0.033	0.046	0.039	0.022	0.022	0.026	0.032	0.099	0.000	0.000	0.000	0.000	0.000	0.000	0.046
40	0.014	0.046	0.077	0.023	0.022	0.019	0.021	0.000	0.107	0.113	0.000	0.000	0.118	0.089	0.046
41	0.020	0.046	0.039	0.022	0.022	0.026	0.032	0.000	0.000	0.000	0.000	0.000	0.118	0.089	0.046
42	0.019	0.047	0.077	0.020	0.022	0.020	0.011	0.099	0.000	0.113	0.000	0.000	0.000	0.089	0.046
43	0.019	0.044	0.039	0.023	0.022	0.026	0.021	0.000	0.000	0.113	0.000	0.000	0.000	0.000	0.046
44	0.037	0.046	0.039	0.023	0.022	0.025	0.016	0.000	0.107	0.000	0.000	0.000	0.118	0.089	0.046
45	0.026	0.047	0.039	0.023	0.022	0.027	0.016	0.000	0.000	0.113	0.000	0.000	0.118	0.089	0.046
46	0.030	0.047	0.039	0.024	0.023	0.026	0.032	0.000	0.107	0.000	0.000	0.000	0.000	0.089	0.046
47	0.033	0.044	0.039	0.023	0.022	0.024	0.032	0.099	0.000	0.113	0.000	0.000	0.118	0.089	0.046
48	0.017	0.044	0.039	0.023	0.023	0.023	0.011	0.000	0.107	0.113	0.066	0.063	0.000	0.000	0.046
49	0.014	0.042	0.039	0.021	0.022	0.028	0.032	0.099	0.000	0.113	0.066	0.063	0.000	0.000	0.046
50	0.017	0.047	0.077	0.023	0.022	0.027	0.021	0.099	0.000	0.113	0.066	0.063	0.000	0.089	0.046
51	0.028	0.044	0.039	0.023	0.022	0.027	0.027	0.099	0.107	0.000	0.000	0.063	0.118	0.089	0.046
52	0.014	0.040	0.077	0.022	0.022	0.029	0.021	0.099	0.107	0.000	0.000	0.000	0.000	0.000	0.046
53	0.022	0.038	0.039	0.022	0.022	0.026	0.021	0.099	0.107	0.000	0.000	0.000	0.118	0.089	0.046
54	0.020	0.035	0.077	0.023	0.022	0.026	0.032	0.099	0.107	0.000	0.000	0.000	0.000	0.089	0.046
55	0.024	0.036	0.077	0.023	0.022	0.027	0.024	0.000	0.000	0.000	0.000	0.000	0.000	0.089	0.046
56	0.019	0.039	0.077	0.022	0.022	0.029	0.025	0.000	0.107	0.000	0.000	0.000	0.000	0.000	0.046
57	0.017	0.038	0.039	0.022	0.022	0.028	0.019	0.099	0.107	0.000	0.000	0.000	0.118	0.000	0.046
58	0.013	0.046	0.077	0.021	0.021	0.026	0.021	0.000	0.000	0.000	0.000	0.000	0.118	0.000	0.046
59	0.010	0.047	0.039	0.018	0.020	0.022	0.021	0.000	0.000	0.000	0.000	0.000	0.000	0.000	0.000
60	0.030	0.037	0.077	0.024	0.022	0.031	0.032	0.099	0.107	0.000	0.000	0.000	0.118	0.089	0.046
61	0.008	0.047	0.077	0.021	0.022	0.027	0.021	0.000	0.107	0.000	0.000	0.000	0.000	0.089	0.046
62	0.014	0.045	0.077	0.022	0.022	0.023	0.021	0.000	0.107	0.113	0.000	0.000	0.118	0.089	0.046
63	0.014	0.046	0.077	0.022	0.022	0.027	0.021	0.000	0.107	0.000	0.000	0.000	0.000	0.089	0.046
64	0.039	0.047	0.077	0.023	0.023	0.033	0.032	0.099	0.107	0.113	0.000	0.000	0.118	0.089	0.046
65	0.027	0.042	0.077	0.024	0.022	0.030	0.032	0.099	0.107	0.000	0.000	0.000	0.118	0.089	0.046
66	0.040	0.047	0.077	0.024	0.023	0.030	0.032	0.099	0.000	0.000	0.000	0.000	0.118	0.089	0.046
67	0.024	0.046	0.077	0.023	0.023	0.034	0.016	0.099	0.107	0.000	0.000	0.000	0.118	0.089	0.046
68	0.005	0.047	0.077	0.024	0.022	0.026	0.021	0.000	0.000	0.000	0.066	0.063	0.000	0.089	0.046
69	0.021	0.047	0.077	0.022	0.022	0.028	0.043	0.099	0.107	0.113	0.066	0.063	0.000	0.089	0.046
70	0.043	0.047	0.077	0.024	0.022	0.032	0.025	0.099	0.107	0.113	0.000	0.000	0.118	0.089	0.046
71	0.046	0.016	0.077	0.024	0.022	0.032	0.044	0.099	0.107	0.113	0.066	0.000	0.118	0.089	0.046
72	0.035	0.037	0.077	0.023	0.022	0.033	0.043	0.000	0.107	0.113	0.000	0.000	0.118	0.089	0.046
73	0.033	0.035	0.077	0.023	0.022	0.034	0.043	0.099	0.107	0.113	0.000	0.000	0.118	0.089	0.046
74	0.039	0.040	0.077	0.024	0.022	0.031	0.021	0.099	0.107	0.000	0.000	0.000	0.118	0.089	0.046
75	0.030	0.038	0.077	0.024	0.022	0.031	0.024	0.000	0.107	0.113	0.000	0.000	0.118	0.089	0.046
76	0.026	0.035	0.077	0.022	0.022	0.030	0.021	0.000	0.000	0.000	0.000	0.000	0.000	0.089	0.046
77	0.048	0.038	0.077	0.023	0.022	0.034	0.021	0.099	0.107	0.113	0.066	0.000	0.118	0.089	0.046
78	0.042	0.045	0.077	0.023	0.022	0.033	0.021	0.099	0.107	0.113	0.000	0.000	0.118	0.089	0.046

**Table 6:** Final ranking of COIVID-19 patients.

Patient No.	S	R	Q	Rank	Patient No.	S	R	Q	Rank
8	0.077	0.035	0.000	1	48	0.575	0.113	0.768	40
3	0.156	0.046	0.116	2	49	0.585	0.113	0.774	41
9	0.171	0.046	0.125	3	32	0.545	0.118	0.784	42
59	0.178	0.047	0.136	4	57	0.555	0.118	0.790	43
1	0.216	0.046	0.153	5	26	0.563	0.118	0.795	44
5	0.279	0.089	0.449	6	45	0.566	0.118	0.797	45
76	0.369	0.089	0.503	7	44	0.569	0.118	0.798	46
55	0.370	0.089	0.503	8	17	0.573	0.118	0.801	47
39	0.365	0.099	0.557	9	21	0.623	0.118	0.831	48
13	0.293	0.107	0.564	10	66	0.625	0.118	0.833	49
6	0.189	0.118	0.568	11	53	0.649	0.118	0.847	50
11	0.304	0.107	0.571	12	50	0.712	0.113	0.851	51
68	0.486	0.089	0.574	13	37	0.661	0.118	0.854	52
20	0.277	0.113	0.588	14	34	0.662	0.118	0.855	53
10	0.336	0.107	0.590	15	25	0.663	0.118	0.856	54
28	0.351	0.107	0.599	16	29	0.666	0.118	0.857	55
19	0.386	0.107	0.621	17	31	0.680	0.118	0.866	56
56	0.387	0.107	0.621	18	38	0.681	0.118	0.867	57
12	0.391	0.107	0.624	19	47	0.683	0.118	0.867	58
2	0.286	0.118	0.627	20	15	0.686	0.118	0.869	59
22	0.347	0.113	0.630	21	40	0.697	0.118	0.876	60
43	0.353	0.113	0.633	22	62	0.698	0.118	0.877	61
18	0.382	0.113	0.651	23	67	0.702	0.118	0.879	62
7	0.349	0.118	0.665	24	16	0.709	0.118	0.884	63
46	0.463	0.107	0.667	25	60	0.713	0.118	0.886	64
61	0.467	0.107	0.670	26	74	0.715	0.118	0.887	65
63	0.473	0.107	0.674	27	65	0.715	0.118	0.887	66
52	0.478	0.107	0.676	28	35	0.715	0.118	0.887	67
4	0.369	0.118	0.677	29	75	0.719	0.118	0.890	68
14	0.385	0.118	0.687	30	51	0.732	0.118	0.897	69
58	0.390	0.118	0.690	31	72	0.744	0.118	0.904	70
36	0.482	0.113	0.712	32	69	0.843	0.113	0.931	71
41	0.461	0.118	0.733	33	27	0.796	0.118	0.936	72
54	0.577	0.107	0.736	34	78	0.835	0.118	0.960	73
24	0.533	0.113	0.743	35	73	0.839	0.118	0.962	74
30	0.542	0.113	0.748	36	70	0.842	0.118	0.964	75
33	0.544	0.113	0.749	37	64	0.847	0.118	0.967	76
23	0.554	0.113	0.755	38	71	0.899	0.118	0.998	77
42	0.564	0.113	0.761	39	77	0.901	0.118	1.000	78

## Data Availability

A real dataset was used in our proposed study that was extracted from References [8,39].

## Appendix A

**Table A1:** The normalized MEM based on the CRITIC method.

	Demographic	Laboratory Findings				Vital Signs		Symptoms					Chronic Conditions		
Patients	Age	Lymphocyte Count	C-reactive Protein	Urea	Creatinine	Oximetry Saturation	Body Temperature	Pleuritic Chest Pain	Nasal Congestion	Cough	Lost Sense of Smell	Lost Sense of Taste	Heart Disease	Diabetes Disease	Cancer
1	0.703	0.287	0.5	0.162	0.069	0.432	0.512	1	1	1	1	1	1	1	0
2	1.000	0.550	1	0.277	0.106	0.750	0.756	1	1	1	1	1	0	0	1
3	0.797	0.248	1	0.050	0.023	0.636	0.878	1	1	1	1	1	1	1	0
4	0.676	0.364	1	0.032	0.032	0.318	1.000	1	1	1	1	1	0	0	0
5	0.568	0.132	1	0.068	0.028	0.250	0.756	1	1	1	1	1	1	0	0
6	0.743	0.264	1	1.000	1.000	0.659	0.756	1	1	1	1	1	0	1	1
7	0.838	0.147	1	0.201	0.042	0.864	1.000	1	1	1	1	1	0	0	0
8	0.892	0.287	1	0.284	0.134	1.000	1.000	1	1	1	1	1	1	1	1
9	0.635	0.628	1	0.101	0.069	0.273	0.512	1	1	1	1	1	1	1	0
10	0.595	0.837	1	0.061	0.005	0.705	0.756	1	0	1	1	1	1	0	0
11	0.635	0.612	1	0.097	0.012	0.636	0.659	1	0	1	1	1	1	0	1
12	0.878	0.000	1	0.237	0.014	0.386	0.268	1	0	1	1	1	1	0	0
13	0.635	0.116	1	0.036	0.014	0.341	0.756	1	0	1	1	1	1	1	0
14	0.338	0.031	0.5	0.014	0.005	0.295	0.268	1	1	1	1	1	0	1	0
15	0.284	0.008	0.5	0.036	0.009	0.205	0.268	0	0	1	1	1	0	0	0
16	0.338	0.372	1	0.050	0.014	0.432	0.756	0	0	0	1	1	0	0	0
17	0.432	0.039	0.5	0.058	0.014	0.341	0.268	1	0	1	1	1	0	0	0
18	0.973	0.008	1	0.230	0.074	0.273	0.756	0	1	0	1	1	1	1	0
19	0.635	0.132	1	0.104	0.032	0.455	0.512	1	0	1	1	1	1	0	0
20	0.905	0.008	1	0.237	0.083	0.545	0.756	1	1	0	1	1	1	1	0
21	0.568	1.000	0.5	0.068	0.028	0.182	0.268	0	0	1	1	1	0	0	0
22	0.905	0.798	1	0.086	0.032	0.432	0.512	1	1	0	1	1	1	0	0
23	0.568	0.186	0.5	0.014	0.000	0.136	0.268	0	1	0	1	1	1	0	0
24	0.635	0.078	0.5	0.086	0.032	0.341	0.756	1	0	0	1	1	1	0	0
25	0.595	0.233	0.5	0.025	0.009	0.273	0.268	0	1	0	1	1	0	0	0
26	0.500	0.295	0.5	0.036	0.019	0.205	0.268	1	0	1	1	1	0	0	0
27	0.770	0.256	0	0.004	0.009	0.386	0.268	0	0	0	1	1	0	0	0
28	0.568	0.109	0.5	0.068	0.028	0.159	0.512	1	0	1	1	1	1	1	0
29	0.635	0.124	0.5	0.007	0.007	0.250	0.512	1	0	0	1	1	0	0	0
30	0.446	0.078	0.5	0.032	0.006	0.318	0.634	0	1	0	1	1	1	0	0
31	0.297	0.101	0.5	0.043	0.009	0.205	0.268	0	0	1	1	1	0	0	0
32	0.324	0.054	0.5	0.058	0.010	0.750	0.512	0	1	1	1	1	0	0	0
33	0.703	0.062	0.5	0.011	0.000	0.432	0.268	0	1	0	1	1	1	0	0
34	0.297	0.054	0.5	0.004	0.005	0.364	0.756	0	1	0	1	1	0	0	0
35	0.392	0.047	0	0.058	0.009	0.250	0.268	0	0	1	1	1	0	0	0
36	0.838	0.070	0	0.104	0.028	0.409	0.512	1	0	0	1	1	1	1	0
37	0.432	0.031	0.5	0.054	0.019	0.295	0.878	1	0	0	1	1	0	0	0
38	0.230	0.132	0.5	0.061	0.023	0.205	0.268	0	0	1	1	1	0	0	0
39	0.297	0.070	0.5	0.083	0.019	0.250	0.268	0	1	1	1	1	1	1	0
40	0.703	0.062	0	0.054	0.009	0.432	0.512	1	0	0	1	1	0	0	0
41	0.581	0.062	0.5	0.104	0.023	0.227	0.268	1	1	1	1	1	0	0	0
42	0.608	0.031	0	0.155	0.009	0.409	0.756	0	1	0	1	1	1	0	0
43	0.595	0.101	0.5	0.061	0.014	0.250	0.512	1	1	0	1	1	1	1	0
44	0.230	0.062	0.5	0.032	0.014	0.273	0.634	1	0	1	1	1	0	0	0
45	0.446	0.047	0.5	0.065	0.019	0.205	0.634	1	1	0	1	1	0	0	0
46	0.365	0.039	0.5	0.025	0.005	0.227	0.268	1	0	1	1	1	1	0	0
47	0.297	0.093	0.5	0.061	0.012	0.295	0.268	0	1	0	1	1	0	0	0
48	0.635	0.101	0.5	0.043	0.000	0.318	0.756	1	0	0	0	0	1	1	0
49	0.703	0.140	0.5	0.129	0.026	0.182	0.268	0	1	0	0	0	1	1	0
50	0.635	0.039	0	0.032	0.019	0.205	0.512	0	1	0	0	0	1	0	0
51	0.419	0.109	0.5	0.043	0.028	0.205	0.390	0	0	1	1	0	0	0	0
52	0.703	0.186	0	0.086	0.023	0.159	0.512	0	0	1	1	1	1	1	0
53	0.541	0.233	0.5	0.097	0.046	0.227	0.512	0	0	1	1	1	0	0	0
54	0.581	0.287	0	0.029	0.032	0.250	0.268	0	0	1	1	1	1	0	0
55	0.486	0.256	0	0.058	0.019	0.205	0.463	1	1	1	1	1	1	0	0
56	0.608	0.194	0	0.101	0.028	0.136	0.439	1	0	1	1	1	1	1	0
57	0.649	0.225	0.5	0.086	0.042	0.182	0.561	0	0	1	1	1	0	1	0
58	0.730	0.062	0	0.115	0.074	0.250	0.512	1	1	1	1	1	0	1	0
59	0.784	0.031	0.5	0.248	0.120	0.364	0.512	1	1	1	1	1	1	1	1
60	0.365	0.248	0	0.022	0.014	0.091	0.268	0	0	1	1	1	0	0	0
61	0.824	0.043	0	0.133	0.028	0.205	0.512	1	0	1	1	1	1	0	0
62	0.716	0.074	0	0.108	0.028	0.318	0.512	1	0	0	1	1	0	0	0
63	0.703	0.054	0	0.079	0.019	0.205	0.512	1	0	1	1	1	1	0	0
64	0.189	0.031	0	0.029	0.005	0.023	0.268	0	0	0	1	1	0	0	0
65	0.432	0.140	0	0.014	0.009	0.114	0.268	0	0	1	1	1	0	0	0
66	0.162	0.039	0	0.018	0.000	0.114	0.268	0	1	1	1	1	0	0	0
67	0.500	0.070	0	0.054	0.005	0.000	0.634	0	0	1	1	1	0	0	0
68	0.905	0.047	0	0.022	0.023	0.250	0.512	1	1	1	0	0	1	0	0
69	0.568	0.039	0	0.094	0.023	0.182	0.024	0	0	0	0	0	1	0	0
70	0.095	0.039	0	0.011	0.019	0.068	0.439	0	0	0	1	1	0	0	0
71	0.041	0.674	0	0.000	0.030	0.068	0.000	0	0	0	0	1	0	0	0
72	0.270	0.240	0	0.043	0.037	0.023	0.024	1	0	0	1	1	0	0	0
73	0.297	0.287	0	0.050	0.042	0.000	0.024	0	0	0	1	1	0	0	0
74	0.176	0.178	0	0.018	0.023	0.091	0.512	0	0	1	1	1	0	0	0
75	0.378	0.217	0	0.000	0.023	0.091	0.463	1	0	0	1	1	0	0	0
76	0.446	0.295	0	0.086	0.042	0.114	0.512	1	1	1	1	1	1	0	0
77	0.000	0.225	0	0.043	0.028	0.000	0.512	0	0	0	0	1	0	0	0
78	0.122	0.085	0	0.029	0.029	0.045	0.512	0	0	0	1	1	0	0	0
Sigma (standard deviation)	0.232	0.201	0.382	0.123	0.113	0.199	0.231	0.502	0.497	0.495	0.288	0.268	0.502	0.439	0.247

## Appendix B

**Table A2:** The correlation coefficient values of the criteria.

	Age	Lymphocyte Count	C-reactive Protein	Urea	Creatinine	Oximetry Saturation	Body Temperature	Pleuritic Chest Pain	Nasal Congestion	Cough	Lost Sense of Smell	Lost Sense of Taste	Heart Disease	Diabetes Disease	Cancer
Age	1.000	0.091	0.397	0.419	0.199	−0.558	0.425	0.471	0.318	0.101	0.055	−0.133	0.512	0.378	0.310
Lymphocyte count	0.091	1.000	0.371	0.044	0.084	−0.189	0.028	0.066	−0.038	0.125	0.003	0.150	0.008	0.378	0.218
C-reactive protein	0.397	0.285	1.000	0.362	0.232	−0.652	0.508	0.339	0.325	0.195	0.227	0.127	0.204	0.245	0.334
Urea	0.419	0.044	0.362	1.000	0.919	−0.457	0.306	0.257	0.271	0.171	0.088	0.060	0.091	0.363	0.630
Creatinine	0.199	0.084	0.232	0.919	1.000	0.284	0.199	0.166	0.197	0.131	0.050	0.050	−0.064	0.284	0.548
Oximetry saturation	0.558	0.189	0.652	0.457	0.284	1.000	0.584	0.374	0.359	0.169	0.187	0.097	0.210	0.194	0.518
Body temperature	0.425	0.028	0.508	0.306	0.199	0.584	1.000	0.401	0.277	0.069	0.158	0.093	0.196	0.239	0.288
Pleuritic chest pain	0.471	0.066	0.339	0.257	0.166	0.374	0.401	1.000	0.116	0.221	0.159	0.119	0.290	0.249	0.242
Nasal congestion	0.318	−0.038	0.325	0.271	0.197	0.359	0.277	0.116	1.000	−0.024	−0.003	−0.045	0.248	0.270	0.200
Cough	0.101	0.125	0.195	0.171	0.131	0.169	0.069	0.221	−0.024	1.000	−0.003	−0.045	0.248	0.270	0.200
Lost sense of smell	0.055	0.003	0.227	0.088	0.050	0.187	0.158	0.159	−0.003	0.285	1.000	0.150	0.040	0.132	0.218
Lost sense of taste	−0.133	0.150	0.127	0.060	0.050	0.097	0.093	0.119	−0.045	0.150	0.751	1.000	−0.159	−0.021	0.082
Heart Disease	0.512	0.008	0.204	0.091	−0.064	0.210	0.196	0.290	0.248	0.040	−0.159	−0.215	1.000	−0.051	0.076
Diabetes Disease	0.378	0.378	0.245	0.363	0.284	0.194	0.239	0.249	0.270	0.132	−0.021	−0.051	0.399	1.000	0.073
Cancer	0.310	0.218	0.334	0.630	0.548	0.518	0.288	0.242	0.200	0.218	0.082	0.076	0.073	0.206	1.000

## Appendix C

**Table A3:** The quantity of information (*C_j_*).

Medical Source	Sigma	Sum	*C_j_*
Age	0.232	11.015	2.550
Lymphocyte count	0.201	13.032	2.621
C-reactive protein	0.382	10.873	4.148
Urea	0.123	10.475	1.293
Creatinine	0.113	10.721	1.213
Oximetry saturation	0.199	9.167	1.827
Body temperature	0.231	10.229	2.359
Pleuritic chest pain	0.502	10.530	5.284
Nasal congestion	0.497	11.529	5.733
Cough	0.495	12.173	6.027
Lost sense of smell	0.288	12.251	3.524
Lost sense of taste	0.268	12.678	3.400
Heart Disease	0.502	12.615	6.329
Diabetes Disease	0.439	10.867	4.776
Cancer	0.247	10.059	2.480
		Sum	53.564
